# Supplementary material for: Empirical methods for controlling false positives and estimating confidence in ChIP-Seq peaks
Source: BMC Bioinformatics. 2008 Dec 5;9:523. doi: 10.1186/1471-2105-9-523 (PMC2628906; doi:10.1186/1471-2105-9-523)
Supplement: Additional File 1 — A variety of html documents from the USeq web site detailing the available applications, their best usage, output file type descriptions, command line menus, etc. [file 1471-2105-9-523-S1.zip › USeqUserGuides/JavaDoc.zip!/JavaDoc/trans/anno/RandomRegions.html]

RandomRegions


|  |  |  |
| --- | --- | --- |
| **Overview** \| **Package** \| **Class** \| **Use** \| **Tree** \| **Deprecated** \| **Index** \| **Help** | |  |
| **PREV CLASS**   **NEXT CLASS** | **FRAMES**    **NO FRAMES**     **All Classes** |  |
| SUMMARY: NESTED \| FIELD \| CONSTR \| METHOD | DETAIL: FIELD \| CONSTR \| METHOD |  |


---


## trans.anno Class RandomRegions

```
java.lang.Object
  trans.anno.RandomRegions
```

---

``` public class RandomRegions extends java.lang.Object ```

Use to generate random regions of a defined length from a file of regions (chrom, start, stop).
No attention is paid to the chromosome so if you want chromosome matching then split the regions file by
chromosome and make a seperate RandomRegions object.

---

**Constructor Summary**

`RandomRegions(java.io.File regionsFile)`

`RandomRegions(java.io.File regionsFile,
java.io.File genomicSequenceDirectory)`


| **Method Summary** | |
| --- | --- |
| `int` | `countLengths(Region[] rs)` |
| `boolean[][]` | `fetchGCContent(Region[] r)`             Converts the Region[] sequences into boolean[]s, everything not g or c are recorded as false. |
| `int[][]` | `fetchRandomCoordinates(int length, int number)`             Given a desired length of sequence, returns multiple random region start stop coordinates. |
| `int[][]` | `fetchRandomCoordinates(int length, int number, double fractionGCContent)`             Given a desired length of sequence, returns multiple random region start stop coordinates. |
| `Region` | `findRandomRegion(int minimumLength)`             Attempt to find a random Region of the appropriate minimum length. |
| `int[]` | `findRandomSegment(Region region, int length)`             Returns a random segment of the appropriate length within the region. |
| `static Region[]` | `loadBinaryCoordinatesAsRegionArray(java.io.File file)`             Reads binary chrom start stop file into Region[] |
| `static Region[]` | `loadWriteBinaryCoordinates(java.io.File regionsFile)`             Looks for a binary version of the file xxx.corr, if found loads binary, otherwise it parses the txt file and then writes the binary for future use. |
| `static void` | `main(java.lang.String[] args)` |
| `void` | `makeRegions(java.io.File regionsFile)`             Parses the regions file but then adds a pointer per base pair length/ divider to the Region[] for every region. |
| `static void` | `saveRegionsAsCoordinatesArray(java.io.File file, Region[] r)`             Writes the chrom start stop of a Region[] as a binary file. |
| `void` | `setGenomicSequenceDirectory(java.io.File genomicSequenceDirectory)` |

**Methods inherited from class java.lang.Object**

`equals, getClass, hashCode, notify, notifyAll, toString, wait, wait, wait`

**Constructor Detail**

### RandomRegions

```
public RandomRegions(java.io.File regionsFile)
```

---


### RandomRegions

```
public RandomRegions(java.io.File regionsFile,
                     java.io.File genomicSequenceDirectory)
```


**Method Detail**

### main

```
public static void main(java.lang.String[] args)
```

---


### loadWriteBinaryCoordinates

```
public static Region[] loadWriteBinaryCoordinates(java.io.File regionsFile)
```

:   Looks for a binary version of the file xxx.corr, if found loads binary, otherwise it parses the txt file
    and then writes the binary for future use.

---


### loadBinaryCoordinatesAsRegionArray

```
public static Region[] loadBinaryCoordinatesAsRegionArray(java.io.File file)
```

:   Reads binary chrom start stop file into Region[]

---


### saveRegionsAsCoordinatesArray

```
public static void saveRegionsAsCoordinatesArray(java.io.File file,
                                                 Region[] r)
```

:   Writes the chrom start stop of a Region[] as a binary file.

---


### fetchRandomCoordinates

```
public int[][] fetchRandomCoordinates(int length,
                                      int number)
```

:   Given a desired length of sequence, returns multiple random region start stop coordinates.

    :   **Returns:**: int[number of random regions][start stop], will return null if random regions of a given length could not be found.

---


### fetchRandomCoordinates

```
public int[][] fetchRandomCoordinates(int length,
                                      int number,
                                      double fractionGCContent)
```

:   Given a desired length of sequence, returns multiple random region start stop coordinates.

    :   **Returns:**: int[number of random regions][start stop], will return null if random regions of a given length could not be found.

---


### findRandomSegment

```
public int[] findRandomSegment(Region region,
                               int length)
```

:   Returns a random segment of the appropriate length within the region.
    Assumes the Region is larger than the length.

---


### findRandomRegion

```
public Region findRandomRegion(int minimumLength)
```

:   Attempt to find a random Region of the appropriate minimum length.
    Will return null after regions.length attempts.

---


### fetchGCContent

```
public boolean[][] fetchGCContent(Region[] r)
```

:   Converts the Region[] sequences into boolean[]s, everything not g or c are recorded as false.
    If end exceeds the length of the chromosome, gc trunkated.

---


### makeRegions

```
public void makeRegions(java.io.File regionsFile)
```

:   Parses the regions file but then adds a pointer per base pair length/ divider to
    the Region[] for every region. This is to prevent bias in randomly selecting
    regions.

---


### countLengths

```
public int countLengths(Region[] rs)
```

---


### setGenomicSequenceDirectory

```
public void setGenomicSequenceDirectory(java.io.File genomicSequenceDirectory)
```


---


|  |  |  |
| --- | --- | --- |
| **Overview** \| **Package** \| **Class** \| **Use** \| **Tree** \| **Deprecated** \| **Index** \| **Help** | |  |
| **PREV CLASS**   **NEXT CLASS** | **FRAMES**    **NO FRAMES**     **All Classes** |  |
| SUMMARY: NESTED \| FIELD \| CONSTR \| METHOD | DETAIL: FIELD \| CONSTR \| METHOD |  |


---
